# rDNA Genetic Imbalance and Nucleolar Chromatin Restructuring Is Induced by Distant Hybridization between *Raphanus sativus* and *Brassica alboglabra*

**DOI:** 10.1371/journal.pone.0117198

**Published:** 2015-02-27

**Authors:** Hong Long, Chunli Chen, Bing Wang, Yanni Feng

**Affiliations:** 1 College of Horticulture and Landscape, Tianjin Agricultural University, Tianjin, China; 2 College of Life Sciences and Technology, Huazhong Agricultural University, Wuhan, Hubei, China; Huazhong university of Science and Technology, CHINA

## Abstract

The expression of rDNA in hybrids inherited from only one progenitor refers to nucleolar dominance. The molecular basis for choosing which genes to silence remains unclear. We report genetic imbalance induced by distant hybridization correlates with formation of rDNA genes (NORs) in the hybrids between *Raphanus sativus* L. and *Brassica alboglabra* Bailey. Moreover, increased CCGG methylation of rDNA in F_1_ hybrids is concomitant with *Raphanus*-derived rDNA gene silencing and rDNA transcriptional inactivity revealed by nucleolar configuration restriction. Newly formed rDNA gene locus occurred through chromosomal in F_1_ hybrids via chromosomal imbalance. NORs are gained *de novo*, lost, and/or transposed in the new genome. Inhibition of methyltransferases leads to changes in nucleolar architecture, implicating a key role of methylation in control of nucleolar dominance and vital nucleolar configuration transition. Our findings suggest that gene imbalance and methylation-related chromatin restructuring is important for rDNA gene silencing that may be crucial for synthesis of specific proteins.

## Introduction

Distant hybridization may occur when different genera (intergeneric hybridization) or different species (interspecific hybridization) are crossed. When successful, it can accelerate gene exchange between diverse genera or species. Since extensive genetic variants can be created during the process, it is a useful tool for researching phylogenesis and genetics [1]. In addition, allopolyploids may be produced, and this polyploidy is a prominent and significant force in plant evolution [2]. Distant hybridization has been applied to rice, wheat, maize, cotton, soybean, and *Brassica* systems, its use has resulted in a number of potential cultivars, varieties, as well as some alien substitution lines, additional lines, and translocation lines [3] [4]. These genetic stocks or lines are useful in plant breeding.

Epigenetic changes occur in allopolyploids that result from distant hybridization events [5] [6]. The resulting doubling of the genome significantly affects gene expression, resulting in epigenetically induced gene silencing [5] [7]. These variations were prominent in genetically unstable F_1_ generations after two different genomes were combined [8]. The most typical epigenetic change in plant distant hybridization is nucleolar dominance. The phenomenon results in cytologically visible changes in chromosome morphology [9], since 45S ribosomal RNA genes (rRNA genes) are inherited from only one progenitor due to the silencing of the other progenitor’s rRNA genes. The molecular basis that determines which genes are silenced remains unclear. Evidence showed that silencing of rRNA genes is related to DNA methylation and histone deacetylation [10]. Inhibition of DNA methylation by aza-deoxycytosine (aza-dC) and of histone deacetylation with trichostatin (TSA) in *Brassica* both prevented nucleolar dominance [11] [12]. Moreover, this gene silencing is a manifestation of rRNA gene dosage control, which is dependent upon the number of active rRNA genes needed by the metabolism of the cell [13]. Small RNAs corresponding to the rRNA gene promoter and intergenic regions also play a role in regulating rRNA gene silencing [10].

Altered chromatic structure may affect many phenotypic changes of eukaryotic cells due to changes in gene expression. Processes involved in the alteration of chromatin are diverse, including post-translational modifications of histone proteins, incorporation of specific histone variants, methylation of DNA, and ATP-dependent chromatin remodeling [14]. In eukaryotic cells, condensed chromosomal DNA (heterochromatin) is one of the key regulating motifs involved in gene silencing. Tandem arrayed repeats of active rRNA genes in the nucleolus display typical characteristics of euchromatin, including histone H3K4 methlation and hyperacetylation of histone H3 and H4. In contrast, the silenced rRNA genes appear as heterochromatin, with characteristics including H3K9 methylation, histone hypoacetylation and DNA methylation [15] [16]. The evidence to date implies that these chromatin–changing functions are important and generate an epigenetic regulatory circuit that is not well understood.

In this paper, we synthesized F_1_ hybrids using embryo rescue, which resulted in distant hybridization between *Raphanus sativus* and *Brassica alboglabra*. We provide cytogenetic evidence for chromosomal changes in both the F_1_ and the subsequent allotetraploid. Furthermore, we described the changes in nucleolar architecture and rDNA chromatin remodeling that are induced by the hybridization and the formation of allopolypoids. We also examined the changes after DNA demethylation, resulting from treatments with methyltransferase inhibitor 5-azacytidine. Our data suggest that chromosomal rearrangements, genetic imbalance, and epigenetic motifs regulate nucleolar structure and chromatin remodeling, and may control rRNA gene expression in intergeneric hybrids.

## Materials and Methods

### Plant materials, field hybridization and embryo rescue

Selfed generations of *Raphanus sativus* cv. HQ-04 (a vegetable radish landrace in Wuhan) and *Brassica alboglabra* were used to synthesize amphidiploid *Raphanobrassica*. Field hybridizations and embryo rescue procedures were performed according to Li *et al*. (2010). The 45S rDNA probe was provided by Prof. Lijia Li, Wuhan University, China.

### Genomic DNA isolation and Southern blot analyses

Seeds of *Raphanus sativus* and *Brassica alboglabra* were surface sterilized with 75% ethanol for 30 seconds, rinsed with sterile water, and then planted in a growth cabinet (Sanyo, Osaka, Japan) with 16 h light at 22℃. Genomic DNA was isolated from fully expanded leaves from each genus, as well as from F_1_ plants that resulted from embryo rescue, using a modified CTAB method [17] and purified by phenol extractions. Quality and quantity of DNA were determined by both gel electrophoresis and spectrometric assays. Southern blots were performed using genomic DNA of the parents and F_1_ hybrids with wheat pTa71 45S rDNA as the probe.

### RNA isolation and RT-PCR

RNA was isolated with Trizol method. Universal primers (p1: 5’-CCCAACTACAGACCAA CTATC-3’; p2: 5’-CTTATGTGTTCACGACTTCCC-3’) were designed using start sites of rDNA transcription in *Raphanus sativus* and *Brassica alboglabra*. RT-PCR was performed with template of cDNA from reverse transcription of total RNA in leaves of *Raphanus sativus, Brassica alboglabra* and F_1_. The reaction system includes template cDNA 4 μl, p1(10μM) 1 μl, p2(10μM) 1 μl, 10×BufferⅡ(including Mg2+) 5 μl, 2.5 mM dNTPs 4 μl, TransTaq HiFi DNA Polymerase 0.5 μl, ddH2O to final volume of 50 μl. Thermal cycles were initiated at 94℃ for 5 min; then proceeded to 40 cycles of 94℃ for 40 s, 54℃ for 40 s, 72℃ for 20 s and finished with 72℃ for 20 s. The amplification products were separated on 1.5% agarose gel electrophoresis with 0.5 μg ml-1 ethidium bromide in 0.5×TBE buffer at 100 V for 3.5–4 h and photographed under ultraviolet light. Sequencing was finished after gel extraction. Comparison of RT-PCR products from F_1_, *Raphanus sativus* and *Brassica alboglabra* was performed with Clustal x to determine nucleolar dominance.

### Cytogenetic preparation

Ovary tissues were used to generate mitotic spreads for in situ hybridization. In brief, ovary materials were successively immersed in saturated para-dichlorobenzene for 3 h, double distilled water for 30 min, mixed with 2.5% (w/v) cellulase and pectinase for 1 h, double distilled water for 20 min and Carnol’s fixation (methanol:acetic acid = 3:1, v/v). Finally, ovaries were squashed on a slide and dried with flame on alcohol burner.

### Fluorescence *in situ* hybridization

FISH was carried out as described by the nonradioactive in situ hybridization application manual provided by Roche (Roche-Applied Science, Penzberg, Germany). Briefly, probes were labeled with Dig-11-dUTP in DIG High Prime (Roche-Applied Science, Penzberg, Germany). Hybridization buffer contained 50% deionized formamide, 2xSSC, 50 mM/L sodium phosphate (pH 7.0), 5% dextran sulfate and 3 ng/uL probe. The buffer was denatured at 98℃ for 10 min before use. Slides with metaphase spreads were treated with 70% deionized formamide in 2x SSC at 68℃ for 2 min, then applied 15 μl denatured hybridization buffer onto the slides, and incubated at 80℃ for 5 min and at 37℃ overnight. The slides were washed with 2x SSC, 30% deionized formamide at 37℃ for 5 min and 2x SSC at 37℃ for 5 min twice. Signals were detected with anti-DIG-fluorescein-conjugate. Metaphase spreads were counterstained with 10 ng/ml DAPI. The epifluorescence signal was observed under a Leica DM4000B LED microscope (Leica Company, Wetzlar, Germany), recorded with Spot Pursuit CCD camera (Sterling Heights, Michigan, USA), and treated with Image-Pro Plus software (Media Cybernetics, Inc., Rockville, Maryland, USA).

### Electron microscopy

Root pole cells were isolated from F_1_ hybrids and the parents. Root pole meristems were obtained from seedlings grown under standard conditions at 27℃ or after treatment with 100 uM 5-azacytidine for 48 h, 60 h and 72 h, respectively. Twenty root tips for each specimen were carefully excised and fixed with 2.5% glutaraldehyde in 0.1mol/L phosphate buffered saline (PBS), pH 7.4, for 2 h at room temperature. After rinsing in double distilled water for 20 min, they were post-fixed in 1% osmium tetroxide for 60 min, then dehydrated with an ethanol/acetone series and embedded in Epon 812. Ultra-thin sections (60–80 nm thick) were stained with uranyl acetate and lead citrate and observed in a Hitachi-7500 transmission electron microscope. More than 20 root pole meristem cells were observed for each specimen.

### Spatial statistical analysis of rDNA in nucleolus

Spatial statistical analysis of rDNA was carried out on transmission electron micrographs using an Image-Pro Plus software (Media Cybernetics, Inc., Rockville, Maryland, USA).

## Results

### F_1_ hybrids obtained through embryo rescue showed intermediate phenotype of the two parents

**Fig 1:**
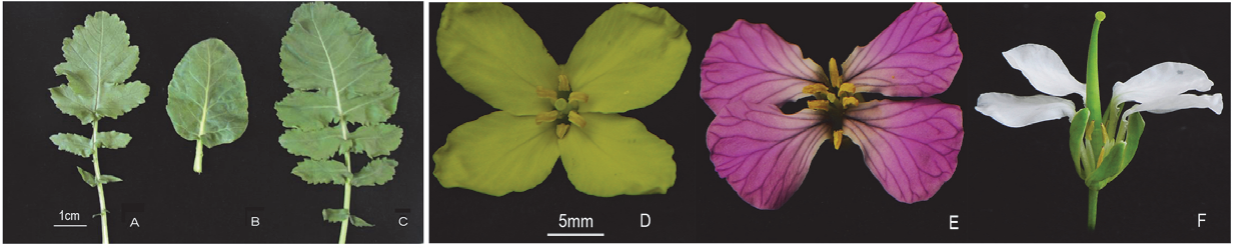
Leaves and flowers from parents and F_1_ plants after embryo rescue. A-C are leaves of *Raphanus*(divided), *Brassica*(indivisus) and F_1_ plants(lobed), respectively. Bar = 1cm. D-F are flowers of *Brassica*(yellow), *Raphanus*(pinch) and F_1_ plants(white). Bar = 5mm.

Through denudation and embryo rescue, six F_1_ hybrids were obtained [8]. The hybrids have similar phenotype. The leaf shape of the F_1_ hybrids was different than the two parents, indicating that these F_1_ plants resulted from hybridization events. The leaf margins of the F_1_ hybrids were lobed, those of the female parents (*Raphanus*) and male parents (*Brassica*) were divided and not divided, respectively (Fig. 1A-C). The flowers of F_1_ hybrids were white and sterile, while those of female parents (*Raphanus*) were pink and male parents (*Brassica*) were yellow, both were fertile (Fig. 1D-F). The F_1_ plants obtained through embryo rescue appeared to be true hybrids, based on the preliminary assessment of these phenotypes. With RAPD amplification, the genetic basis of the hybrids was confirmed [8].

### Chromosomal Cross hybridization in the hybrids demonstrated genetic imbalance

**Fig 2:**
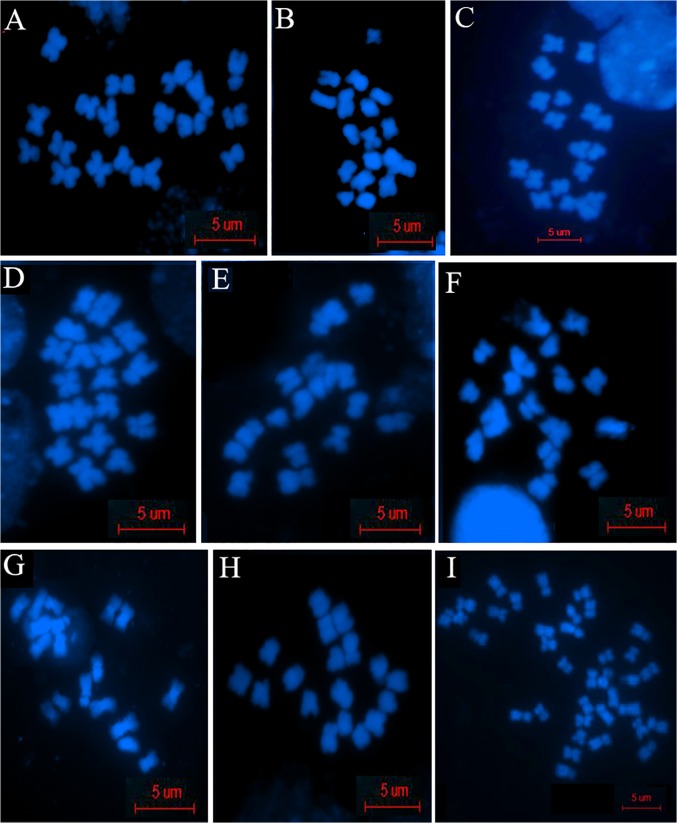
Chromosome number of spreading ovary cells with different materials. A-F showed spreading chromosomes of 6 plants of F_1_ hybrids. G, H, I were those from *Raphanus, Brassica* and F_10_, respectively.

**Fig 3:**
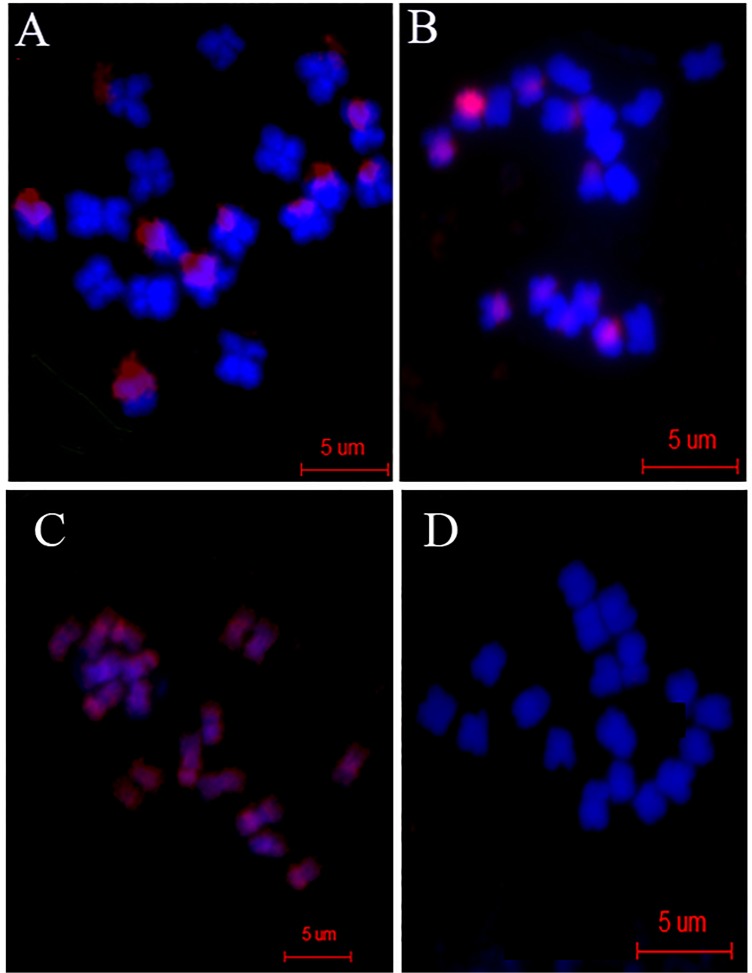
Chromosome constitution of F_1_ hybrids (A,B), *Raphanus* (C) and *Brassica* (D) with Bio-11-dUTP labeled *Raphanus* genomic DNA as a probe. Across 18 chromosomes of F_1_, some were 11R+7B (A), 11 were from *Raphanus* (11R) and 7 from *Brassica* (7B), while the others were 10R+8B (Fig. 3B). C and D were for FISH specificity.

Ovary cells from F_1_ and F_10_ [8] were used for chromosome spreads to evaluate chromosomal status in hybrids and allopolyploids. The chromosome numbers of *Raphanus* (Fig. 2G), *Brassica* (Fig. 2H) and 6 F_1_ hybrids (Fig. 2A-F) were 18 for each, while that of allopolyploid of F_10_ was 36 (Fig. 2I). We analyzed the genomic structure of *Raphanus, Brassica* and F_1_ hybrid by using fluorescence in situ hybridization (FISH) with Bio-11-dUTP labeled *Raphanus* genomic DNA as a probe. By hybridizing the *Raphanus* probe to six hybrids, it was showed that the signals of 18 chromosomes of the F_1_ were not equally derived from *Raphanus* (9R) and *Brassica* (9B), but 4 F_1_ plants were 11R+7B (Fig. 3A) and the other 2 plants were 10R+8B (Fig. 3B), indicating that chromosomal partial hybridization in F_1_ hybrids resulted in a chromosomal imbalance. Although chromosomal imbalance emerged, these FISH data also certified the genetic basis of the hybrids from a cytological aspect.

### rDNA gene silencing induced by the distant hybridization and its retention

**Fig 4:**
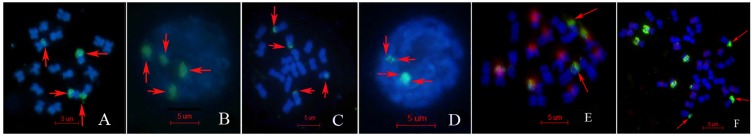
45S rDNA in situ hybridization of *Raphanus* (A and B) and *Brassica* (C and D). A and C for chromosome spread, B and D for nucleus. Arrows show signals. E for F_1_ and F for F_10_ double FISH. Arrows show signals from *Brassica*.

In order to identify 45S rRNA gene [nucleolus organizer region (NOR)] clusters in the parents, fluorescence *in situ* hybridization was conducted with Dig-11-dUTP labeled 45S rDNA as a probe on the metaphase-spread chromosomes of both *Raphanus* and *Brassica* (Fig. 4A-D). Hybridizations of 45S rDNA probes to *Raphanus* and *Brassica* metaphase chromosomes revealed four NOR (Fig. 4A, green signals) signals in each set of 18 chromosomes of the two genomes. Both pairs of NORs are similar in size and are terminally located on the short arms of acrocentric chromosomes (Fig. 4A, C). With Dig-11-dUTP labeled 45S rDNA and Bio-11-dUTP labeled *Raphanus* genomic DNA as probes for dual fluorescence *in situ* hybridization, we further investigated the source of rDNA genes in F_1_ hybrid and F_10_ generations (Fig. 4E, F). FISH analysis of F_1_ hybrid revealed 5 NORs, all on acrocentric chromosomes (Fig. 4E). Among them, two *Brassica*-derived NORs (green signals only) and 3 *Raphanus*-derived NORs (green+red signals) were discriminated by the dual FISH. Six F_1_ hybrid plants were apparently haploid prior to genome doubling (2n = 2x = 18), suggesting that there should be four NORs. However, five NORs were detected instead of 4. Furthermore, one would predict that there should be eight NORs in F_10_ generation after the genome was duplicated (2n = 4x = 36), four from paternal and four from maternal sources. Three NORs were apparently derived from *Brassica* (green signals only), five from *Raphanus* (green+red signals) in the F_10_ generation. These results indicated that rDNA genes from the parents were not lost in the hybridization. An additional *Raphanus*-derived NOR apparently arose *de novo* in the F_1_ generation. During the course of genome doubling, NOR numbers could stabilize (the sum of the two numbers from the parents, as expected), however, the structure was rearranged at an uneven distribution rate, indicating that rDNA genes may shift from one genome to the other after doubling took place. rDNA may act like a transposable-element or jumping gene as it plays a role in ribosome biosynthesis.

**Fig 5:**
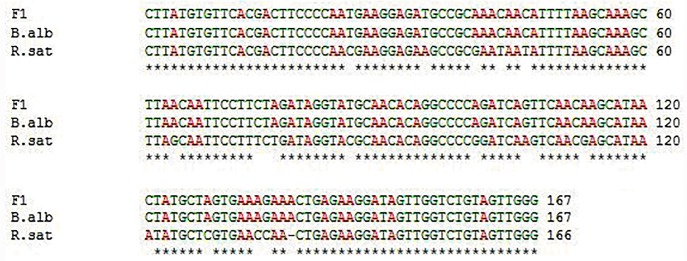
Sequencing of F_1_ hybrid (F1), *Brassica* (B.alb) and *Raphanus*(R.sat). ﹡ stands for locus with the same bases.

We compared transcription sequences to determine which parental set of rRNA genes is silenced in the genetic hybrids. The transcribed spacer of rRNA gene in *Brassica alboglabra* was cloned using RT-PCR through homologous sequencing from *Brassica oleracea*. After a comparison of results from multiple clones of transcribed sequence with the known sequence of the transcribed spacer of the rRNA gene in *Raphanus sativus*, the DNA sequence of F_1_ hybrid was the same as those of *Brassica alboglabra* (Fig. 5), indicating that the hybridization induced nucleolar dominance. rRNA gene from *Brassica alboglabra* expressed, while the other (from *Raphanus sativus*) was silenced.

### rDNA methylation changes occurred during the distant hybridization

**Fig 6:**
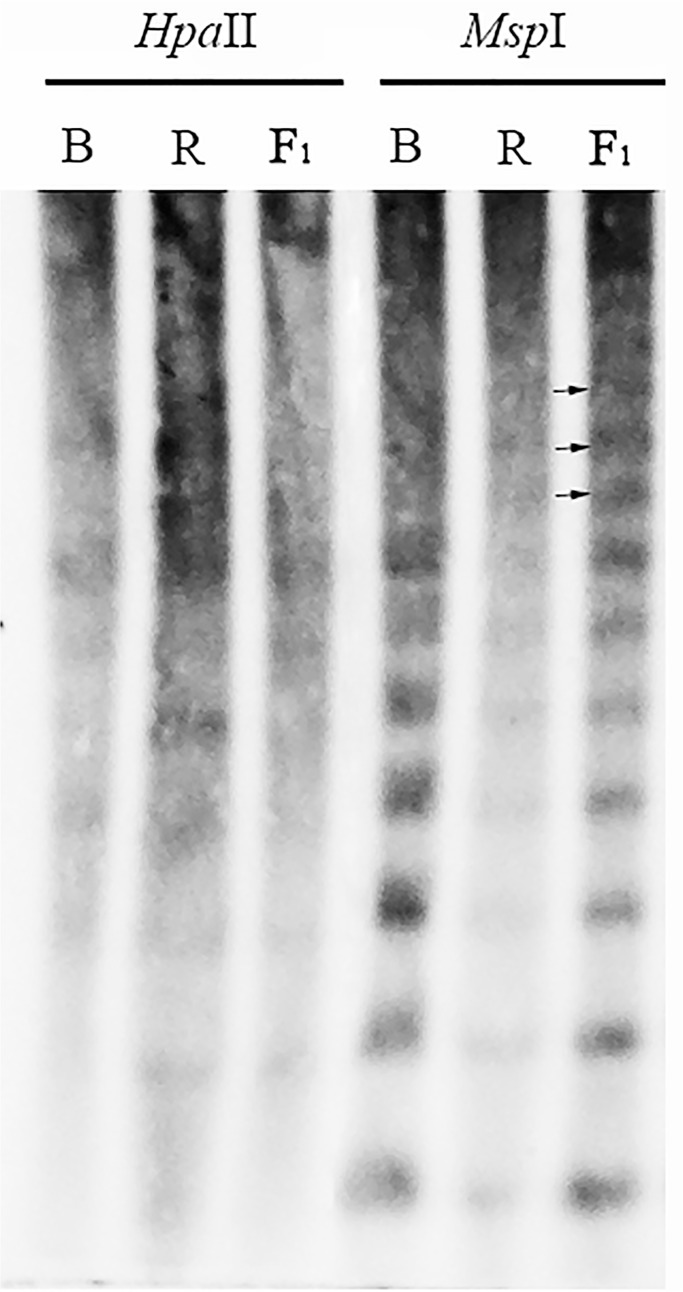
rDNA methylation changes in the formation of hybrids. Southern blot was performed with 45S rDNA as a probe. Arrows show new bands of lane 6 compared with lane 4 and lane 5.

Evidence suggested that epigenetic changes occurred during the formation of F_1_ hybrids that resulted from distant hybridizations [18] [19] [20] [21], so we examined the methylation of rDNA in this hybrid. Using 45S rDNA as probes, Southern blots were performed using genomic DNA of parents and F_1_ hybrids after digestion with a pair of DNA methylation sensitive endonucleases, *Msp*I and *Hpa*II. *Msp*I and *Hpa*II are isoschizomers [22] that recognize the same restriction site (CCGG) but have different sensitivities to certain methylation states of cytosines. *Hpa*II will not cut if either of the cytosine (internal or external) is methylated, whereas, *Msp*I will not cut only if the external cytosine is methylated [23]. Thus, for a given DNA sample, the methylation of the internal or external cytosine at the assayed CCGG sites can be unequivocally distinguished [22] [24] using these two restriction enzymes. Southern blot results using 45S rDNA as a probe showed that there were multiple methylation events on the internal cytosine of CCGG (Fig. 6). Meanwhile, in comparison with its *Brassica* and *Raphanus* parents, the F_1_ hybrids exhibited DNA methylation changes in at least 5 of the 12 sequences analyzed. Hypermethylation (increase in methylation level) changes were also observed in F_1_ hybrids (new bands were detected on lane 6 compared with lane 4 and lane 5, Fig. 6, arrows). Furthermore, these hypermethylation events were due to increases in the internal cytosine on CCGG. These findings are consistent with the 45S rDNA silencing status.

### Nucleolar configuration restructuring reflects rDNA transcriptional variation from active to inactive states after hybridization

**Fig 7:**
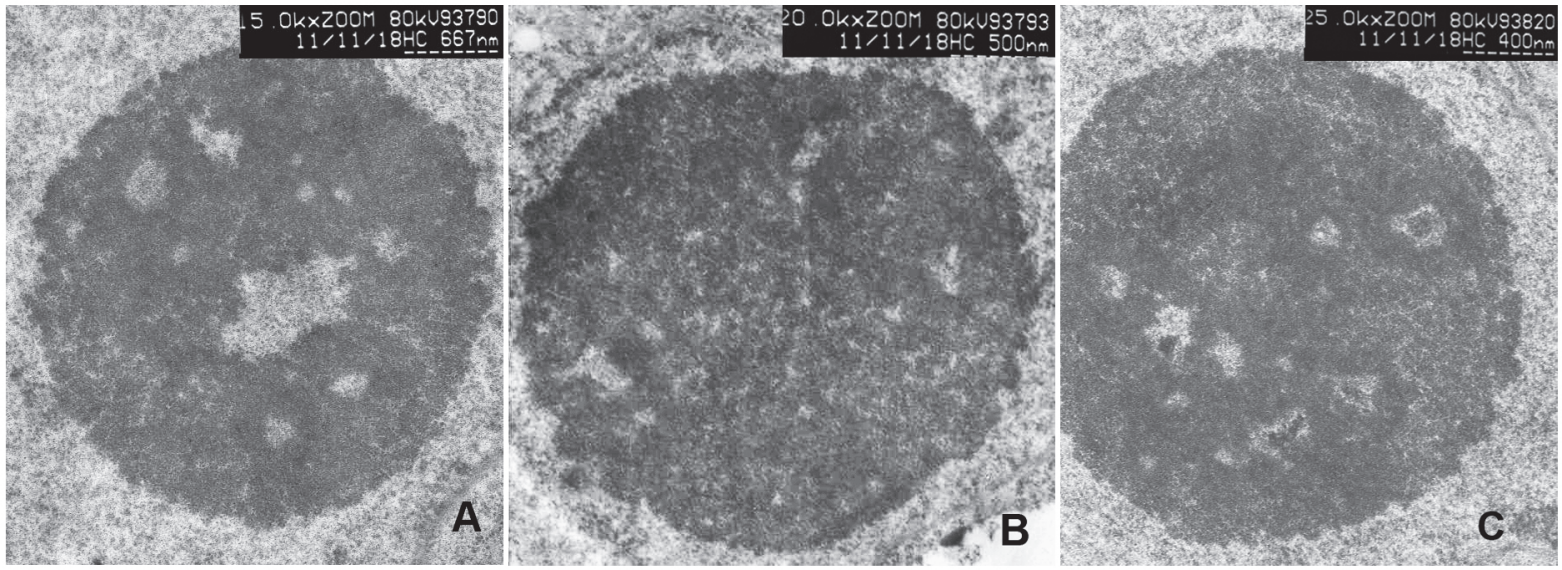
Conventional staining of ultrathin sections of *Raphanus* (A), *Brassica* (B) and F_1_ plants (C, blocks show heterogeneous chromatin in FCs).

Since epigenetic changes occurred in the formation of F_1_ hybrids, we compared the nucleolar architecture of F_1_ hybrid and its parents to further test epigenetic effects on rDNA transcriptional states. The nucleolar architecture of the two parents, *Raphanus sativus* L. and *Brassica alboglabra* Bailey, showed a typical nucleolar under the electron microscope (Fig. 7A, 7B). Conventionally stained, nucleoli of the two species appear as granular components (GCs) surrounding dense fibrillar components (DFCs) around pale fibrillar centers (FCs) (Fig. 7A). The FC is the lowest electron dense region and is filled with a substance presumed to be chromatin [25]. The DFC has the highest electron density. The granular component is located between the DFC and the border of the nucleolus (Fig. 7A). Nucleolus-Vacuoles can often be detected on cross-sections (Fig. 7A). The nucleolar architecture of F_1_ hybrids is similar to that of the parents. However, some structural variations were detected. In the nucleoli, the chromatin in the FCs assumed one of the two states of condensation, condensed into clumps (heterogeneous FC) or dispersed and diffuse (homogeneous FC). The heterogeneous FC represents transcriptionally inactive states of rDNA, while homogeneous FC represents transcriptionally active states of rDNA [26]. Here we show that the nucleolar architecture of the F_1_ hybrids has more heterogeneous than homogeneous FCs compared with its parents (Fig. 7A-C). Statistical analyses of the heterogeneous/homogeneous chromatin in FC of F_1_ hybrid and its parents *Brassica* and *Raphanus* are 64.80%, 35.97% and 32.36%, respectively (Table 1 and Table 2 in S1 File). These results indicate that distant hybridization induces variation of rDNA from transcriptionally active to inactive states, by changing the configuration of the nucleolus. The mechanism of these processes is unknown.

### Nucleolar architecture changed in the F_1_ hybrids after treatment with 5-azacytidine

**Fig 8:**
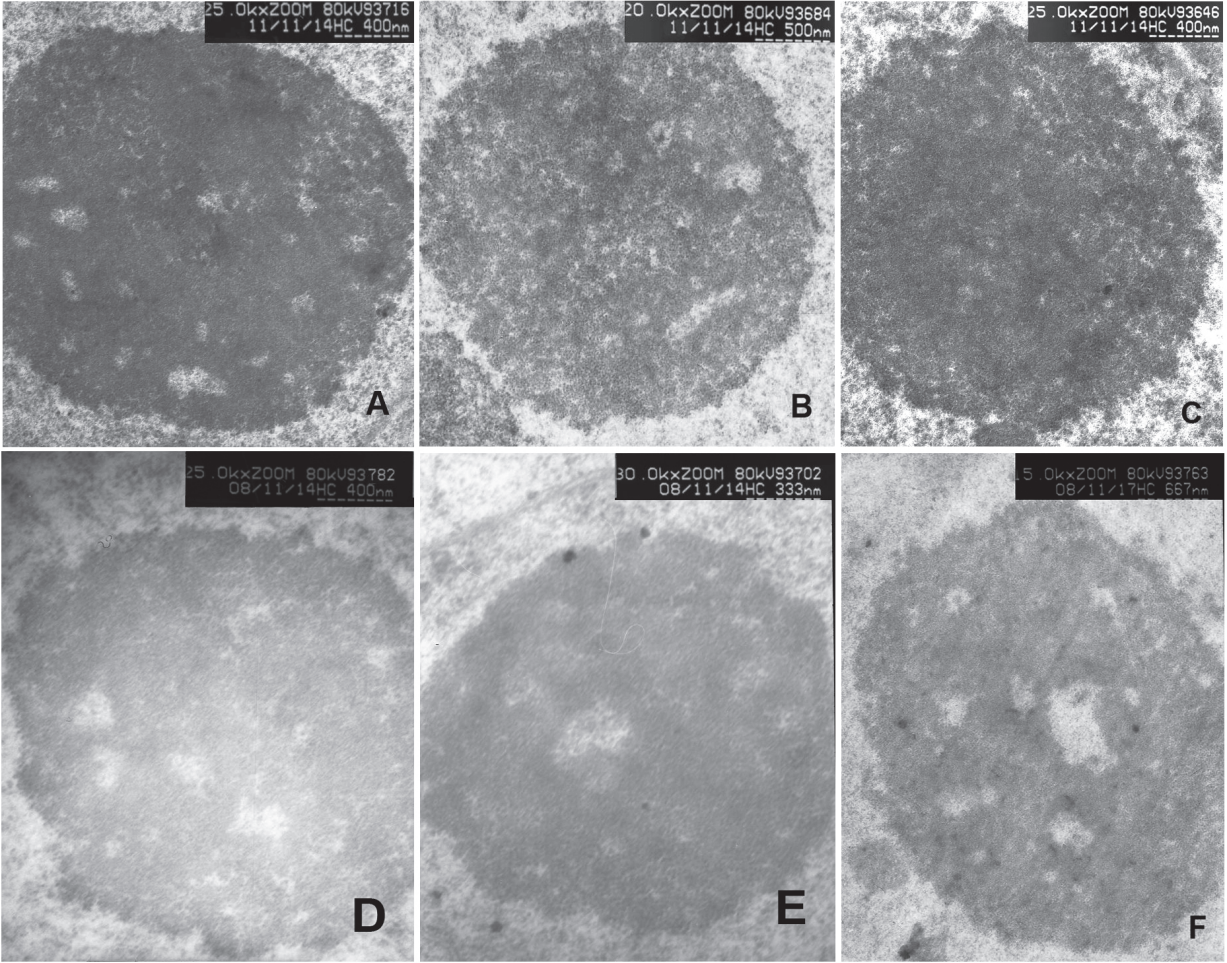
Conventional staining of ultrathin sections of F_1_ plants after treatment with 5-azacytidine for 48 h (A), 60 h (B) and 72 h (C). D-F are controls of A-C, respectively, without treatment of 5-azacytidine.

In order to determine the effects of DNA methylation on nucleolar organization, we compared the nucleolar structure of F_1_ hybrids using electron microscopy before and after treatment with 5-azacytidine. The drug 5-azacytidine (5-AC) is a structurally modified cytosine analog that is an inhibitor of methyltransferases and can be incorporated into the DNA during replication, which leads to the hypomethylation of genomic DNA. The results showed that compared with the untreated controls, the nucleolar architecture in the F_1_ hybrids was more prominent after a 48 h treatment with 5-azacytidine. There was a decrease in the area of the DFC region and an increase in the number of FCs (Fig. 8A). In addition, the ratio of heterogeneous/homogeneous chromatin in FCs decreased. After a 60 h treatment, the trend was more prominent (Fig. 8B), and there was a gradual disintegration of nucleolar architecture, including FC, DFC, and GC. After a 72 h treatment, the nucleolus was uniform, and did not have a distinguishable structure (Fig. 8C). These results indicated that rDNA methylation caused heterogeneity in chromatin, while demethylation led to a decrease in heterogeneous chromatin. Considering the process of forming F_1_ hybrids, we concluded that rDNA methylation may be one of the major causes of rDNA gene silencing.

## Discussion

The processes by which two genomes adapt to coexist within the same nucleus are complex and can differ markedly between species [27]. With respect to interspecific hybridization, much attention has been focused on understanding what happens when divergent genomes are brought together [26]. Gene silencing and/or elimination was the common accepted consequence of both interspecific hybridization and genome duplication. However, the mechanisms underlying the regulation of gene silencing and/or elimination are unclear. Buggs *et al*. examined gene existence and expression in the allotetraploid hybrid *Tragopogon miscellus* [28]. They found that across 10 homeologous gene pairs in 57 natural hybrids, 3.2% of loci had been rendered undetectable in the genomic DNA, suggesting that there was a loss of one parental gene copy. Further analyses of seven of these homeologous pairs using cDNA as a template showed that expression of one parental gene copy had been lost in 6.8% of loci. These results indicated that both gene elimination and silencing happened in the hybrids.

Distant hybridization is the first step towards forming new allopolyploids. About 70% of flowering plants have undergone at least one whole-genome duplication event during their evolutionary history [29], and many of these polyploid genomes eventually returned to a diploid-like state either through a loss or divergence of duplicated genes.

Gene elimination and /or silencing are some of the mechanisms that result in rearranged genes. As part of these processes, the phenomena of rDNA gene silencing in the hybrids (nucleolar dominance) has received attention for a long time. Since rDNA codes for ribosomal RNA, and is assembled into ribosomes that translate mRNA to produce proteins, it is important to elucidate the biological roles of rDNA in hybrids. The effects of rDNA silencing can be recovered in the generations after the hybridization event, as changes in DNA cytosine methylation and histone deacetylation occur, which affect the silencing process [10] [12] [15] [30]. We previously reported on the distant hybridization and amphidiploid formation between *Raphanus sativus* L. and *Brassica alboglabra* Bailey, and showed that the chromosome numbers were unstable and that cytosine DNA methylation occurred on a whole-genome scale [8]. In this work, we continued our genetic analyses of these hybrids and provide additional information about the methylation states of rDNA. Like those observed on a genomic scale, cytosine methylation was also detected in rDNA, with a pattern of hypermethylation. The hybridization also induced nucleolar dominance. The rRNA gene from *Brassica alboglabra* was expressed, while the one from *Raphanus sativus* was silenced. These results indicate that DNA methylated states in rRNA genes may play a role in rDNA gene silencing in this system of distant hybridization between two genera.

Although nucleolar dominance occurs in interspecific hybrids of plants, invertebrates, frogs, flies, fish, and mammals [31] [32], its mechanisms are not fully understood. Evidence showed that the nucleolus only forms at NORs when the rRNA genes are active [33]. NORs that are active during interphase remain relatively decondensed at metaphase, forming the so-called secondary constrictions, while inactive NORs are fully condensed at metaphase. In this paper, our results showed that rDNA transcription changed from the active to the inactive state as observed by using electron microscopy. Moreover, these changes could be returned to their original state after treatment with methyltransferase inhibitors (5-azacytidine), suggesting that an rDNA methylation event was caused by heterogeneity in F_1_ chromatin.

The eukaryotic nucleus represents a complex arrangement of heterochromatic and euchromatic domains, each with their specific nuclear functions. Although the distribution and localization of rDNA in the nucleolus are still controversial, it has been shown that rDNA are mainly located at the periphery of FCs and at the border between FCs and DFCs [34] [35]. After treatment with the demethyltransferase 5-azacytidine, the structure and configuration of nucleoli varied with the level of demethylation. These results indicate from the structural aspect that DNA methylation and demethylation plays a key role in maintaining the stable configuration of nucleolus. With the formation of F_1_ hybrids, DNA methylation act as a factor in controlling the state of DNA activity, resulting in nucleolar dominance.

A chromosomal structural change on the whole genome level is another possibility to explain the event of nucleolar dominance. The nucleolus is a research model used to study how chromatin-regulated transcription relates to nuclear organization. Evidences of nucleolar dominance in plants suggest that rDNA organization and activity are controlled by a combination of DNA methylation, histone modification, and chromatin remodeling [33]. Previous study shows that loss, gain, and or rearrangement of NORs and 5S loci occur in the early generations after formation of the allotetraploid *Arabidopsis suecica* genome and can give rise to alternative chromosomal changes [36]. Their observations suggest that chromosomal alterations in natural and synthetic *A. suecica* follow certain trends, with specific loci that tend to be unstable. However, the consequences of the instability are unpredictable, as demonstrated by the variability in *Arabidopsis thaliana*-derived NOR numbers and chromosomal positions in neoallotetraploids [36]. Homoeologous chromosomes were reported in resynthesized *Brassica napus* allopolyploids. Changes in copy number of individual chromosomes were detected in the S_0:1_ generation and increased in subsequent generations, whereas the mean chromosome number among lines was approximately the same [37]. In this paper, our results show that as chromosomes of the *Raphanus* and *Brassica* are partially hybridizing, chromosomal imbalance occurred, with more chromosome segments from *Raphanus* (11R or 10R) and fewer from *Brassica* (7B or 8B). Likewise, there are five 45S rDNA signals in F_1_ hybrids, two of which are *Brassica*-derived and three of which are *Raphanus*-derived. After genome doubling in the F_10_ generation, 8 hybrid signals were detected, three of which were *Brassica*-derived and five of which were *Raphanus*-derived. More rRNA gene signals from *Raphanus*, both in the genetically labile F_1_ and stable F_10_ generations, were detected than those in *Brassica*. Considering that rRNA genes from *Raphanus* were expressed and those from *Brassica* were silenced, we suggest that gene dosage was not the key factor in controlling rRNA gene expression, and that chromosomal remodeling and rearrangement during distant hybridization and polypolid formation were predominant.

Somatic cells of multicellular organisms are genetically identical, yet they may differ completely in their nuclear organization and gene expression patterns [38]. We recently found that genomic shock cause may result in a labile status of chromosome numbers in the early generations (F_4_-F_8_) of distantly hybridized *Raphanus sativus* L. and *Brassica alboglabra* Bailey, resulting in the formation of mixoploids with 2n = 27–38. After several generations, mixoploids gradually turned to euploids through the formation of neo-chromosomes or chromosome elimination events. The possible cytological mechanisms underlining these hybrid generations are unknown [8].

We also explored rRNA gene expression patterns with this experimental system. rRNA gene silencing was detected in the hybrids, which was apparent as epigenetic alteration in the form of cytosine DNA methylation patterns. Moreover, genetic imbalance at the cytogenetic chromosome level and chromosome remodeling at the ultrastructural configuration level were also detected in the hybrids and the F_10_ generation. Although the causes for nucleolar chromatin remodeling in F_1_ hybrids remain unclear, these configuration changes may be related with methylation-induced rDNA transcriptional variation from active to inactive states after hybridization. We suggest that intergeneric hybridizations result in complex genetic and epigenetic alterations that may lead to a reconfiguration after hybridization occurs between two diploid species. rDNA gene silencing and nucleolar dominance, induced by intergeneric distant hybridization, is one of the gene silencing phenomena that occur in joined nuclei.

## Supporting Information

S1 File Supporting Tables. Table 1. Ratios of heterogeneous/homogeneous chromatin in Fibrilla Center of F_1_ hybrid. Table 2. Ratios of heterogeneous/homogeneous chromatin in Fibrilla Center of *Brassica* and *Raphanus* parents.
(DOC)
