# Supplementary material for: rDNA Genetic Imbalance and Nucleolar Chromatin Restructuring Is Induced by Distant Hybridization between Raphanus sativus and Brassica alboglabra
Source: PLoS One. 2015 Feb 27;10(2):e0117198. doi: 10.1371/journal.pone.0117198 (PMC4344237; doi:10.1371/journal.pone.0117198)
Supplement: S1 File — Table 1. Ratios of heterogeneous/homogeneous chromatin in Fibrilla Center of F1 hybrid. Table 2. Ratios of heterogeneous/homogeneous chromatin in Fibrilla Center of Brassica and Raphanus parents. (DOC) [file pone.0117198.s001.doc]

Table 1 Heterogeneous/homogeneous chromatin in FC of F_1_ hybrid

| Samples | No. of FCs | Heterogeneous FCs | Homogeneous FCs | heterogeneous/homogeneous |
| --- | --- | --- | --- | --- |
| 121028 | 8 | 6 | 2 | 75.00% |
| 111105 | 9 | 7 | 2 | 77.78% |
| 100930 | 7 | 4 | 3 | 57.14% |
| 111027 | 6 | 4 | 2 | 66.67% |
| 111008 | 8 | 5 | 3 | 62.50% |
| 120207 | 9 | 6 | 3 | 66.67% |
| 120321 | 10 | 8 | 2 | 80.00% |
| 111216 | 8 | 4 | 4 | 50.00% |
| 120403 | 7 | 4 | 3 | 57.14% |
| 120513 | 9 | 5 | 4 | 55.56% |
| 120709 | 8 | 5 | 3 | 62.50% |
| 121006 | 9 | 6 | 3 | 66.67% |
| average |  |  |  | 64.80% |

Table 2 Heterogeneous/homogeneous chromatin in FC of *Brassica* and *Raphanus* parents

| Samples | No. of FCs | Hetero- FCs | Homo FCs | hetero/homo | Samples | No. of FCs | Hetero- FCs | Homo- FCs | hetero/homo |
| --- | --- | --- | --- | --- | --- | --- | --- | --- | --- |
|  |  | Brassica |  |  |  |  | Raphanus |  |  |
| 121026 | 4 | 1 | 3 | 25.00% | 121029 | 2 | 1 | 1 | 50.00% |
| 111106 | 3 | 1 | 2 | 33.33% | 111103 | 3 | 1 | 2 | 33.33% |
| 100929 | 4 | 2 | 2 | 50.00% | 100924 | 4 | 1 | 3 | 25.00% |
| 111023 | 2 | 1 | 1 | 50.00% | 111028 | 5 | 1 | 4 | 20.00% |
| 111013 | 5 | 1 | 4 | 20.00% | 111011 | 5 | 2 | 3 | 40.00% |
| 120209 | 4 | 1 | 3 | 25.00% | 120210 | 3 | 1 | 2 | 33.33% |
| 120320 | 3 | 2 | 1 | 66.67% | 120322 | 4 | 1 | 3 | 25.00% |
| 111219 | 3 | 1 | 2 | 33.33% | 111217 | 3 | 1 | 2 | 33.33% |
| 120406 | 2 | 1 | 1 | 50.00% | 120404 | 4 | 1 | 3 | 25.00% |
| 120515 | 3 | 1 | 2 | 33.33% | 120516 | 5 | 1 | 4 | 20.00% |
| 120715 | 5 | 1 | 4 | 20.00% | 120713 | 3 | 1 | 2 | 33.33% |
| 121007 | 4 | 1 | 3 | 25.00% | 121003 | 4 | 2 | 2 | 50.00% |
| average |  |  |  | 35.97% |  |  |  |  | 32.36% |
